# Lipopolysaccharide-induced alterations in barrier integrity and inflammatory signaling in porcine small intestinal organoids

**DOI:** 10.1371/journal.pone.0359086

**Published:** 2026-10-01

**Authors:** Sumin Park, Boram Lee, Dahye Kim, Sheet Sunirmal, Min Gook Lee, Haesun Lee, Sun A. Ock, Jae Gyu Yoo

**Affiliations:** 1 Animal Biotechnology and Genomics Division, National Institute of Animal Science, Rural Development Administration, Wanju, Jeonbuk, Republic of Korea; 2 Department of Bio-medical Analysis, Bio Campus of Korea Polytechnics, Nonsan, Republic of Korea; 3 Department of Companion Animal Business & Science, Jeonju University, Jeonju, Republic of Korea; University of Arizona, College of Medicine-Phoenix, UNITED STATES OF AMERICA

## Abstract

Inflammation-associated diseases in pigs impair the intestinal tract and impair its function, thereby posing a major threat to their health and productivity. However, current *in vitro* cell models fail to fully replicate porcine physiological conditions. In this study, we aimed to develop a novel inflammation model using porcine small intestinal organoids derived from adult jejunum stimulated with lipopolysaccharide (LPS). The organoids were exposed to LPS to induce inflammation, and the responses were evaluated by quantifying cytokine mRNA expression, assessing intestinal epithelial barrier integrity, and analyzing changes in the expression and localization of proteins involved in nutrient transport, receptor signaling, and enteroendocrine function using quantitative real-time PCR and immunofluorescence staining. Transcriptome analysis was performed to identify LPS-responsive differentially expressed genes and enriched pathways in porcine small intestinal organoids. LPS stimulation effectively induced inflammation in porcine small intestinal organoids, as evidenced by increased TLR4 and cytokine gene expression, compromised epithelial barrier integrity, and altered expression and localization of proteins involved in nutrient transport, bile acid signaling, and enteroendocrine function. Furthermore, transcriptome profiling revealed LPS-induced broad transcriptional changes in porcine small intestinal organoids, including enrichment of immune- and inflammation-related pathways, as well as functional categories associated with barrier maintenance, membrane function, and adaptation to inflammatory stress. Collectively, these findings demonstrate that porcine small intestinal organoids provide a physiologically relevant *in vitro* model for studying intestinal inflammation. This model offers a valuable platform for investigating the mechanisms underlying porcine inflammatory bowel diseases and for screening novel therapeutic agents, supporting the 3Rs principle by potentially reducing reliance on animal testing in translational research.

## Introduction

The porcine small intestine is highly responsive to infectious agents and environmental stressors [1]. As the primary site of nutrient absorption and a major immune organ, inflammation directly impairs its growth and productivity [2]. Major small intestinal inflammatory diseases include enteritis caused by porcine epidemic diarrhea virus, rotavirus, enterotoxigenic *Escherichia coli*, and *Salmonella* spp., which lead to severe diarrhea and dehydration, increasing mortality [3,4]. These conditions involve chronic or recurrent inflammation driven by complex mechanisms, such as dysbiosis, impaired mucosal barrier function, and immune hyperactivation [1,5]. Peyer’s patches in the jejunum and ileum serve as key sites for antigen recognition and immune cell activation in pigs [6]. Accordingly, studying porcine small intestinal inflammatory diseases is essential for improving feeding efficiency and survival rates as well as for elucidating intestinal immune mechanisms and developing models to evaluate novel drugs and vaccines. The need for research on inflammatory diseases using porcine small intestinal organoids (pSIOs) is being increasingly emphasized [7–9].

Lipopolysaccharide (LPS), a major component of the cell walls of Gram-negative bacteria, is a potent pro-inflammatory molecule that exacerbates intestinal inflammatory diseases and impairs barrier function [10,11]. Understanding LPS-induced intestinal inflammation and barrier dysfunction is essential for elucidating the physiological and pathological mechanisms of intestinal diseases. LPS-induced inflammation has been studied using porcine intestinal epithelial cell lines [12–16] or pig models [17–19]; however, both models have limitations. Cell lines are easy to culture and partially reproduce species-specific responses but cannot fully reproduce interactions among multiple intestinal cell types [20–22] and may accumulate genetic and morphological alterations during long-term culture [23–25]. In contrast, *in vivo* pig models more accurately reflect actual physiological responses but are constrained by substantial ethical and financial burdens, large inter-individual variability, and challenges in monitoring specific signaling pathways and cell-cell interactions [26–29].

To overcome these limitations, organoid models have recently gained significant attention. Organoids are three-dimensional (3D) *in vitro* models that replicate native organ structure and function and are widely used in disease modeling, drug testing, and nutritional research [7,23,30]. Small intestinal organoids are of particular interest because they accurately recapitulate the structure, cellular composition, and physiological functions of the native small intestine, enabling the study of inflammatory responses in a physiologically relevant context. They enable precise analysis of cellular interactions and immune responses, providing valuable insights into complex physiological phenomena, such as inflammation [31–33]. pSIOs are particularly valuable because of their strong physiological similarity to the human intestine [8,31,34]. Accordingly, organoid models serve as advanced *in vitro* models that complement the limitations of cell lines and animal models while preserving organ-specific structure and function [20,35,36]. Furthermore, organoids support reproducible, long-term experiments while maintaining species-specific characteristics across various applications [8,23,37,38].

Recent studies have established porcine intestinal organoids as physiologically relevant models for investigating enteric viral infections, host–pathogen interactions, and intestinal epithelial biology [39–41]. Furthermore, two-dimensional organoid-derived monolayer systems have been developed to facilitate barrier function assays and pathogen infection studies [42,43]. However, comprehensive characterization of LPS-induced inflammatory responses in porcine small intestinal organoids, including epithelial barrier disruption, nutrient transporter and receptor regulation, enteroendocrine responses, and global transcriptomic alterations, remains limited. To address this gap, the present study aimed to establish a porcine small intestinal organoid-based inflammatory model that integrates molecular, functional, and transcriptomic analyses to provide a comprehensive platform for investigating intestinal inflammation and evaluating potential therapeutic interventions.

In this study, pSIOs were treated with LPS to establish an intestinal inflammatory model. We analyzed transcriptomic and functional changes, including the expression of inflammation-related genes and the expression and localization of barrier-associated proteins, nutrient transporters, receptors, and enteroendocrine hormones. Collectively, these analyses enabled a comprehensive characterization of LPS-induced inflammatory responses in pSIOs and demonstrated the utility of this model as a physiologically relevant in vitro platform for investigating porcine intestinal inflammation and evaluating potential therapeutic interventions.

## Materials and methods

### Experimental design and animals

An LPS-induced pSIO model was established using 10-month-old adult pigs. All animal procedures were approved by the Institutional Animal Care and Use Committee of the National Institute of Animal Science (NIAS 2022−0569). Animals were euthanized by intravenous administration of T61 to minimize animal suffering. No anesthesia or analgesia was administered because tissue collection was performed only after euthanasia. Small intestinal tissues were collected immediately after euthanasia. Crypts isolated from porcine small intestines were three-dimensionally cultured in Matrigel® domes to generate organoids (Fig 1A). Mature pSIOs were then exposed to LPS (Sigma-Aldrich, St. Louis, MO, USA) to induce an inflammatory response. Preliminary dose-response experiments were conducted using 5, 10, 20, and 30 μM LPS to identify the appropriate LPS concentration. The 5 and 10 μM concentrations induced minimal inflammatory responses with no apparent morphological changes, whereas 30 μM caused severe structural disruption accompanied by extensive cell death. In contrast, 20 μM reproducibly induced inflammatory responses with only partial morphological alterations and without extensive cell death. Therefore, 20 μM was selected for all subsequent experiments. pSIOs were cultured in IntestiCult™ medium supplemented with 20 μM LPS for 24 h at 37 °C in a 5% CO_2_ atmosphere. Control pSIOs were maintained under identical conditions without LPS treatment (Fig 1B). Treated pSIOs were analyzed for gene expression, cell viability, and protein expression using quantitative real-time PCR (qRT-PCR), 4′,6-diamidino-2-phenylindole (DAPI)/propidium iodide (PI) staining, immunofluorescence analysis, and transcriptome analysis (Fig 1C).

**Fig 1:**
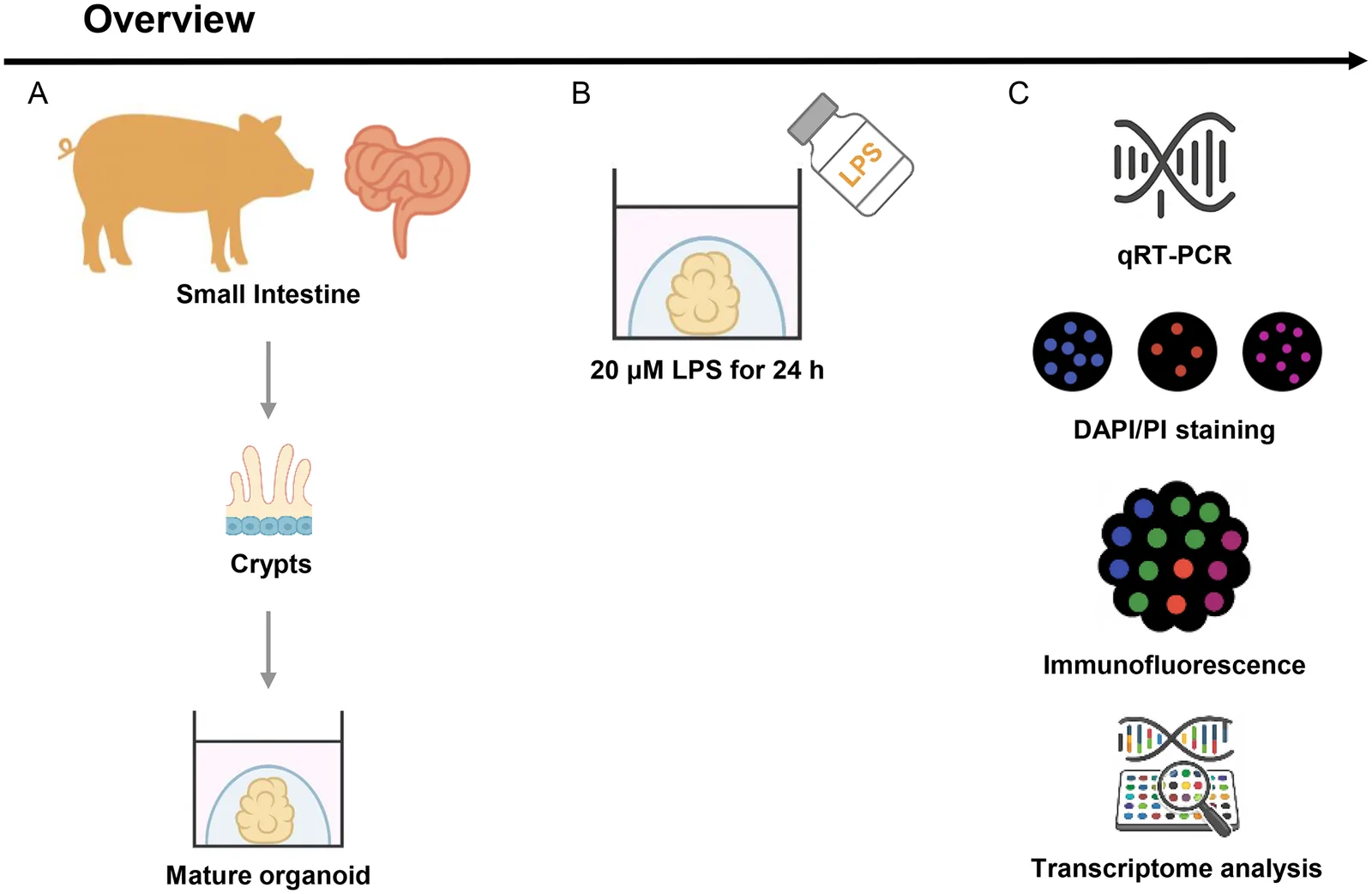
Experimental design overview. (A) Porcine small intestinal crypts were embedded in Matrigel domes and cultured to establish small intestinal organoids. (B) Mature organoids were treated with 20 μM lipopolysaccharide (LPS) for 24 h to induce inflammation. (C) Treated organoids were analyzed using qRT-PCR, DAPI and PI staining, and immunofluorescence to assess gene expression, cell viability, and protein expression.

### 3D culture of pSIOs

Porcine small intestinal crypts were isolated from 10-month-old pigs as previously described [44]. The isolated crypts were counted using an inverted microscope, and approximately 140–150 crypts were mixed with Matrigel® (Corning Incorporated, Corning, NY, USA) at a 1:1 ratio to form domes. The Matrigel domes containing the mixture were dispensed into the center of preheated 24-well plates and allowed to solidify at 37 °C for 15 min. Subsequently, 1 mL of IntestiCult™ Organoid Growth Medium Human Basal Medium (Stemcell Technologies Inc., Vancouver, BC, Canada) was added to each well, and the cultures were maintained at 37 °C in a 5% CO_2_ atmosphere. Mature small intestinal organoids were subcultured approximately once per week.

### RNA extraction and cDNA synthesis

pSIOs and ear-derived fibroblasts were lysed using the RNeasy Mini Kit (Qiagen, Hilden, NRW, Germany). RNA was extracted according to the manufacturer’s instructions. RNA concentration and purity were measured using an ND-2000 Spectrophotometer (Thermo Fisher Scientific, MA, USA). A total of 500 ng of RNA was reverse transcribed into cDNA using the Omniscript RT Kit (QIAGEN GmbH, Hilden, Germany). The 20 µL reaction mixture contained 2 µL of 10 × RT buffer, 2 µL of dNTP mix (5 mM each), 2 μL of oligo(dT) primer (10 µM), 1 μL of RNase inhibitor (10 units/µL), 1 μL of Omniscript Reverse Transcriptase, RNase-free water, and template RNA. All procedures were performed on ice. Reverse transcription was performed at 37 °C for 60 min. The synthesized cDNA was used immediately for qRT-PCR analysis or stored at −20 °C.

### qRT-PCR

Quantitative RT-PCR was performed to compare intestinal epithelial and stem cell marker expression among porcine ear fibroblasts (PEFs) and small intestinal organoids and to analyze inflammatory cytokine expression in LPS-treated organoids.

Each 20 µL reaction contained 10 µL of 2 × SYBR Green Master Mix, 0.5 µL each of forward and reverse primers (10 µM), 1 µL of cDNA template, and 8 µL of RNase-free water. qRT-PCR was performed using SYBR® Green PCR Master Mix (Applied Biosystems, Foster City, CA, USA) on a QuantStudio 3 Real-Time PCR Instrument (Thermo Fisher Scientific) with the following conditions: 95 °C for 10 min, followed by 40 cycles of 95 °C for 15 s and 60 °C for 1 min. Specificity was confirmed by melting curve analysis. For each experiment, organoids were independently cultured, treated, and subjected to RNA extraction, with each independently processed culture considered one biological replicate (n = 3). For each biological replicate, qRT-PCR was performed using five technical replicates. Relative expression levels were calculated using the 2^-ΔΔCt^ method with GAPDH as the endogenous control based on the mean Ct values of the technical replicates. Primer sequences are listed in S1 Table.

### Apoptosis analysis (DAPI/PI staining)

Apoptosis was assessed using DAPI/PI staining. LPS-treated pSIOs were washed with phosphate-buffered saline (PBS; Thermo Fisher Scientific) and fixed with 4% paraformaldehyde (Sigma-Aldrich) for 15 min. The fixed organoids were washed thrice with PBS and stained with 1 μg/mL PI (Sigma-Aldrich) for 10 min at room temperature in the dark. The stained organoids were washed with PBS, mounted on glass slides using VECTASHIELD® Antifade Mounting Medium with DAPI (Vector Laboratories, Burlingame, CA, USA), and nuclear staining was performed simultaneously. Samples were imaged using a confocal laser scanning microscope (AX Series; Nikon Corp., Tokyo, Japan) to assess cell death. DAPI/PI staining was performed using three independent biological replicates (n = 3), and at least five organoids were analyzed for each LPS concentration in each biological replicate.

### Immunofluorescence staining

LPS-treated pSIOs were washed with PBS, fixed with 4% paraformaldehyde for 15 min, and permeabilized with 0.1% Triton X-100 (Sigma-Aldrich) for 10 min. Organoids were blocked with 5% bovine serum albumin (Sigma-Aldrich) for 1 h at room temperature. They were incubated overnight at 4 °C with the following primary antibodies (diluted 1:200): Toll-like receptor 4 (TLR4) monoclonal (Thermo Fisher Scientific), epithelial cadherin (E-cadherin) monoclonal (BD Transduction Laboratories™, San Jose, CA, USA), zonula occludens-1 (ZO-1; Tight Junction Protein 1, TJP1) monoclonal (Santa Cruz Biotechnology™, Dallas, TX, USA), sodium-glucose cotransporter 1 (SGLT1) polyclonal (Novus Biologicals, Centennial, CO, USA), peptide transporter 1 (PEPT1) polyclonal (Bioss Antibodies, Woburn, MA, USA), glucose transporter 2 (GLUT2) polyclonal (Novus Biologicals), glucagon-like peptide-1 (GLP-1) polyclonal (MyBioSource, San Diego, CA, USA), and G-protein-coupled bile acid receptor 1 (TGR5) polyclonal (Abcam, Cambridge, MA, USA) antibodies. After washing with PBS, the organoids were incubated for 1 h at room temperature with secondary antibodies (diluted 1:500) as follows: Alexa Fluor® 647-conjugated donkey anti-rat IgG H&L, Alexa Fluor® 647-conjugated goat anti-mouse IgM mu chain, Alexa Fluor® 488-conjugated goat anti-rabbit IgG H&L (all from Abcam, Cambridge, UK), Alexa Fluor™ 488-conjugated goat anti-mouse IgG2b, and Alexa Fluor® 568-conjugated goat anti-rabbit IgG (both from Thermo Fisher Scientific, Waltham, MA, USA). After staining, organoids were mounted on glass slides using VECTASHIELD® Antifade Mounting Medium containing DAPI. Fluorescent signals were imaged using a confocal laser scanning microscope (Nikon Corp.). Immunofluorescence staining was performed using three independent biological replicates (n = 3), and at least five organoids were analyzed for each treatment condition in each biological replicate.

### RNA sequencing data processing and quantification

For RNA sequencing, total RNA extracted from three organoid wells derived from the same organoid preparation was pooled to generate a single RNA sample for each experimental group (n = 1 per group). Therefore, RNA sequencing was performed without independent biological replicates. The Illumina NovaSeq 6000 platform was used to generate paired-end reads. Raw BCL files were converted to FASTQ format using bcl2fastq. Read quality assessment and trimming were performed using FastQC (v0.11) and Trimmomatic (v0.39) in paired-end mode by trimming ambiguous N bases and filtering reads with a Phred quality score < 20. Clean reads were aligned to the Sus scrofa reference genome (Sscrofa12.1) using HISAT2 (v2.2.1). Gene-level counts were quantified from aligned BAM files using featureCounts (Subread package v2.0.1), and the resulting count matrix was used for downstream analyses.

### Differential gene expression analysis

Differential expression between control and LPS-treated pSIOs was analyzed using the edgeR package in R. Raw gene-level read counts were filtered to remove lowly expressed genes and normalized using the trimmed mean of M-values (TMM) method. Because the experiment included only one biological sample per condition (n = 1), variance estimation from biological replicates was not possible; therefore, differential expression was evaluated under the negative binomial model implemented in edgeR using a fixed dispersion value of 0.1. Raw p-values were adjusted for multiple testing using the Benjamini–Hochberg procedure. Candidate DEGs were defined as those with a log2 fold-change ≥ 1, and genes with a p-value ≤ 0.05 were considered statistically significant. These DEGs were further used for downstream analysis.

### Functional and pathway enrichment analyses of DEGs

To interpret the biological relevance of LPS-responsive transcriptional changes in pSIOs, gene ontology (GO) enrichment analysis, including biological process (BP), molecular function (MF), and cellular component (CC), and Kyoto encyclopedia of genes and genomes (KEGG) pathway enrichment analysis were performed using candidate DEGs identified by edgeR (p-value ≤ 0.05 and log2 fold-change ≥ 1). The DEGs were submitted to DAVID (Database for Annotation, Visualization and Integrated Discovery) for functional annotation. The top 10 significantly enriched GO terms and KEGG pathways were selected for visualization. Pathway plots were generated using the clusterProfiler R package.

### Statistical analysis

Statistical analyses were performed using GraphPad Prism (version 5.0; GraphPad Software, San Diego, CA, USA). Data are presented as the mean ± standard deviation (SD) from at least three independent biological replicates, unless otherwise stated. Statistical significance between two groups was determined using an unpaired Student’s t-test. Differences were considered statistically significant at *p < 0.05, **p < 0.01, or ***p < 0.001.

## Results

### Characterization of porcine intestinal organoids

Gene expression profiles were compared between pEFs and pSIOs using qRT-PCR. Intestinal epithelial markers (villin 1 [*VIL1*], lysozyme [*LYZ*], and chromogranin A [*CHGA*]), intestinal stem cell markers (*LGR5, OLFM4,* and *SOX9*), and the proliferation-related marker *MKI67* were significantly upregulated in pSIOs compared with pEFs (*p* < 0.05; Fig 2A–C, D–G).

**Fig 2:**
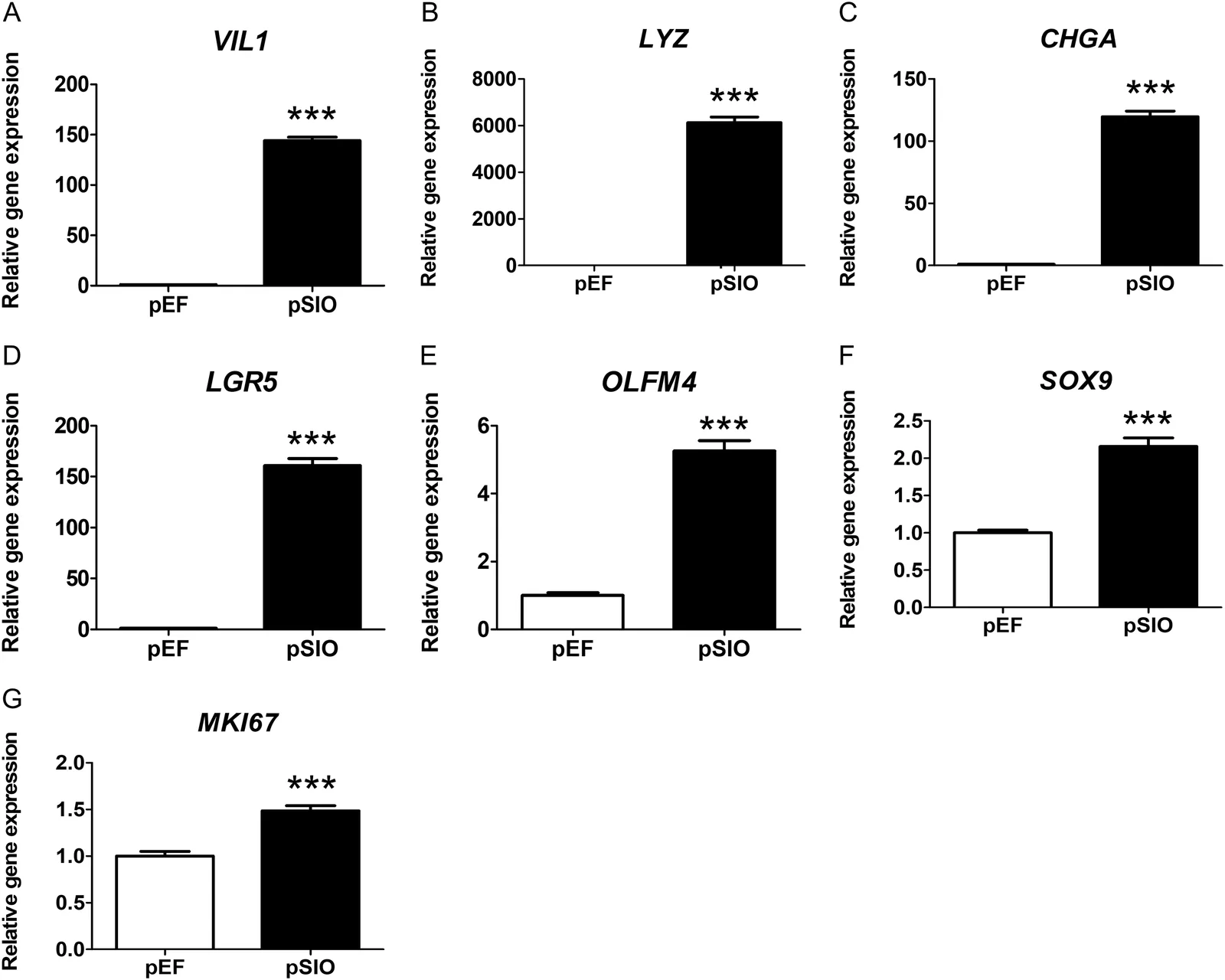
Characterization of porcine small intestinal organoids (pSIOs). Relative mRNA expression in porcine ear fibroblasts (pEFs) and pSIOs was analyzed using qRT-PCR. Intestinal epithelial markers (*VIL1*, *LYZ*, and *CHGA*) (A–C), intestinal stem cell markers (*LGR5*, *OLFM4*, and *SOX9*), and proliferation marker *MKI67* (D–G) were significantly upregulated in pSIOs compared with pEFs. Data are presented as the mean ± SD from three biological replicates (n = 3); each biological replicate was analyzed using five technical replicates. ***p < 0.001.

### Apoptosis and barrier protein expression changes in pSIOs following LPS treatment

Phase-contrast microscopy revealed pronounced structural collapse, core darkening, and fragmentation in LPS-treated pSIOs compared with controls (Fig 3A). After LPS treatment (5, 10, 20, and 30 μM), DAPI/PI staining was performed. Red fluorescence intensity increased with LPS concentration, indicating a dose-dependent increase in apoptosis (Fig 3B). To verify epithelial barrier integrity, immunofluorescence staining for E-cadherin was performed in pSIOs (Fig 3C). In the control group, E-cadherin was strongly and continuously expressed along the epithelial cell membrane, whereas the 20 μM LPS-treated group showed disrupted membrane localization and structural disorganization of E-cadherin. Consistently, qRT-PCR analysis showed that *CDH1* and *TJP1* expression decreased to 0.359-fold (*p* < 0.001) and 0.483-fold (*p* < 0.001) compared to the control (0 μM), respectively, in the 20 μM LPS-treated group.

**Fig 3:**
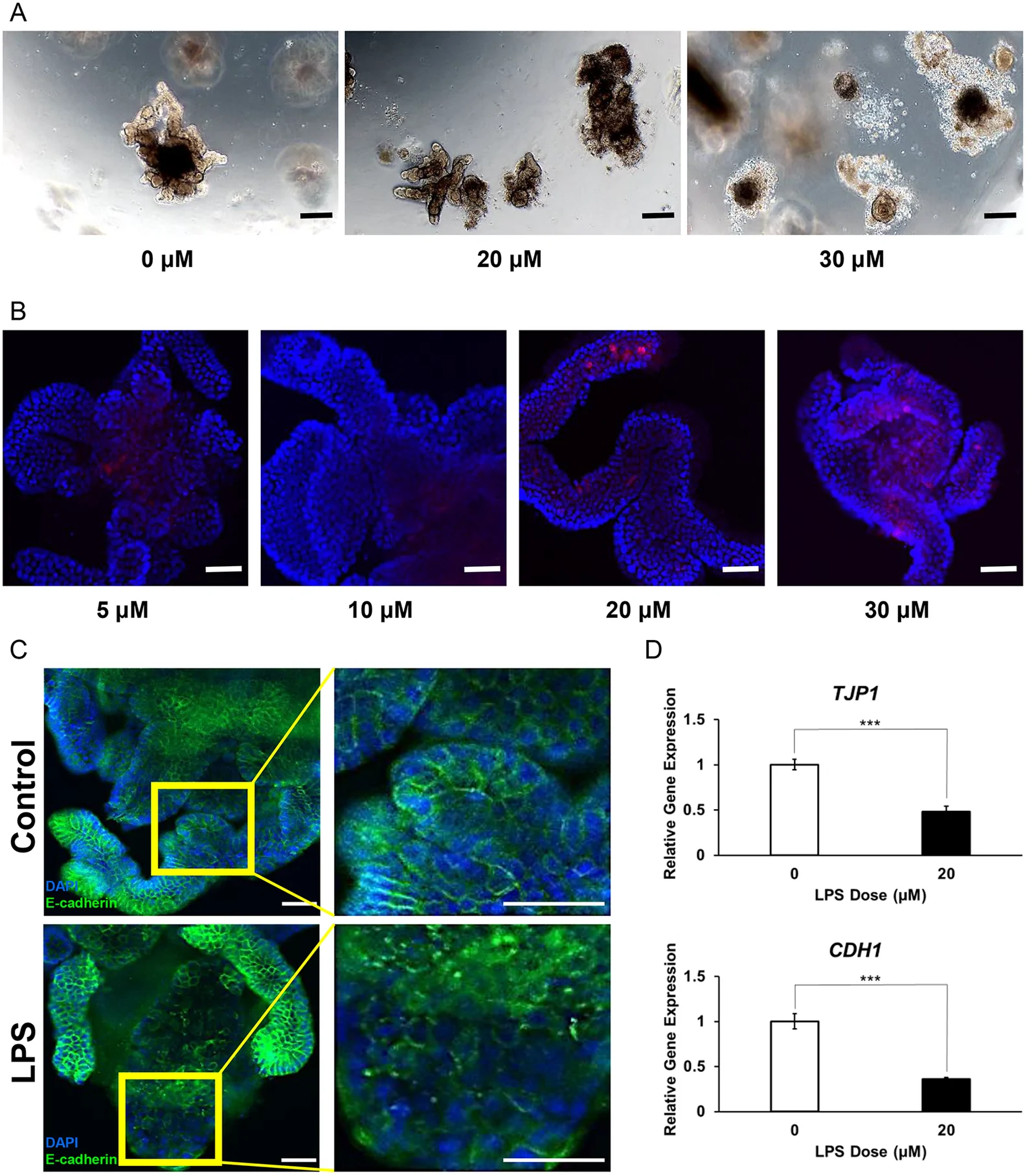
LPS-induced apoptosis and epithelial barrier disruption in pSIOs. (A) Phase-contrast images of pSIOs treated with LPS (0, 20, and 30 μM) showing structural disruption, core darkening, and fragmentation at higher concentrations compared with controls. Scale bars: 200 μm. (B) DAPI (blue) and PI (red) staining of pSIOs treated with LPS (5, 10, 20, and 30 μM) showing a dose-dependent increase in apoptosis. Images are representative of three independent biological replicates (n = 3), with at least five organoids analyzed for each LPS concentration in each biological replicate. Scale bars: 200 μm. (C) Representative immunofluorescence images of E-cadherin (green) and DAPI (blue) staining of pSIOs showing reduced and discontinuous E-cadherin expression in LPS-treated organoids compared with controls. Enlarged views of the yellow boxed regions are shown. Low-magnification (200 μm) images and enlarged views (50 μm) are shown in 3C. Images are representative of three independent biological replicates (n = 3), with at least five organoids analyzed for each treatment condition in each biological replicate. (D) Relative gene expression levels of *CDH1* and *TJP1* in pSIOs treated with LPS (20 μM) compared with controls (0 μM), as determined by qRT-PCR. LPS treatment significantly decreased the expression of *CDH1* and *TJP1*, indicating disruption of epithelial barrier integrity. Data are presented as the mean ± SD from three biological replicates (n = 3); each biological replicate was analyzed using five technical replicates. ***p < 0.001.

### LPS-induced TLR4 and inflammatory cytokine gene expression in pSIOs

To evaluate the inflammatory responsiveness of pSIOs, organoids were treated with LPS (20 μM), and the TLR4 protein and cytokine gene expression were analyzed. Immunofluorescence staining showed TLR4 expression in both the control and LPS-treated groups, with significantly higher fluorescence intensity in the LPS-treated group (Fig 4A). To further characterize the inflammatory response, the mRNA expression levels of pro- and anti-inflammatory cytokines were analyzed by qRT-PCR (Fig 4B). The pro-inflammatory cytokines, *tumor necrosis factor-alpha (TNF-*α), *interleukin (IL)-1β,* and *IL-8* (p < 0.01) and anti-inflammatory cytokines*, IL-4* and *IL-10* (p < 0.05) were significantly upregulated in the LPS-treated organoids, showing 2.61-fold, 3.61-fold, 3.65-fold, 12.15-fold, and 3.40-fold increases, respectively. In contrast, *transforming growth factor beta* (*TGF-β*) expression increased 1.41-fold but did not reach statistical significance.

**Fig 4:**
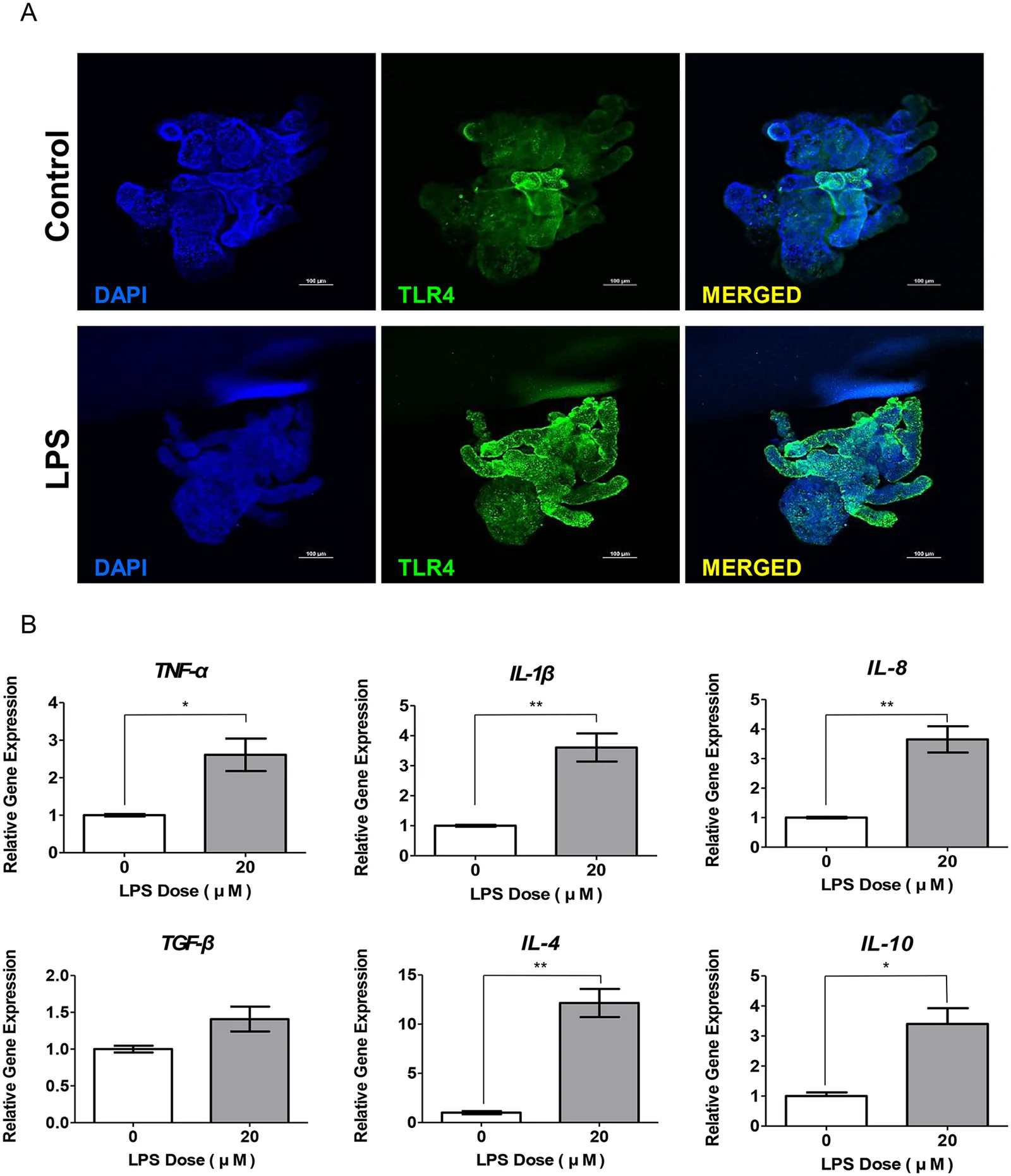
Cytokine gene expression changes in pSIOs following LPS treatment. (A) Immunofluorescence staining of TLR4 in pSIOs after LPS (20 μM) treatment. Nuclei were stained with DAPI (blue), and TLR4 expression is shown in green. Representative images suggest higher TLR4 immunofluorescence in the LPS-treated group than in the control group. Images are representative of three independent biological replicates (n = 3), with at least five organoids analyzed for each treatment condition in each biological replicate. Scale bar = 100 μm. (B) qRT-PCR analysis of pro- and anti-inflammatory cytokines in pSIOs after LPS (20 μM) exposure. Pro-inflammatory cytokines *TNF-α*, *IL-1β*, and *IL-8* were significantly upregulated in the LPS-treated group compared with the control group. Anti-inflammatory cytokines *IL-4* and *IL-10* also showed significantly increased expression, whereas *TGF-β* expression was increased but did not reach statistical significance following LPS stimulation. Data are presented as the mean ± SD from three biological replicates (n = 3); each biological replicate was analyzed using five technical replicates. *p < 0.05, **p < 0.01.

### Changes in metabolic, endocrine, and receptor-related expression in pSIOs following LPS treatment

The pSIOs used in this study represent a basal-out model, in which the basolateral surface of intestinal epithelial cells is exposed to the culture medium. Following 20 μM LPS treatment, immunofluorescence analysis revealed alterations in proteins related to nutrient transport, endocrine function, and receptor signaling (Fig 5A). Consistently, qPCR demonstrated significant changes in the mRNA expression levels of *SLC5A1* (SGLT1), *SLC15A1* (PEPT1), *SLC2A2* (GLUT2), *GPBAR1* (TGR5), and *GCG* (GLP-1) (Fig 5B).

**Fig 5:**
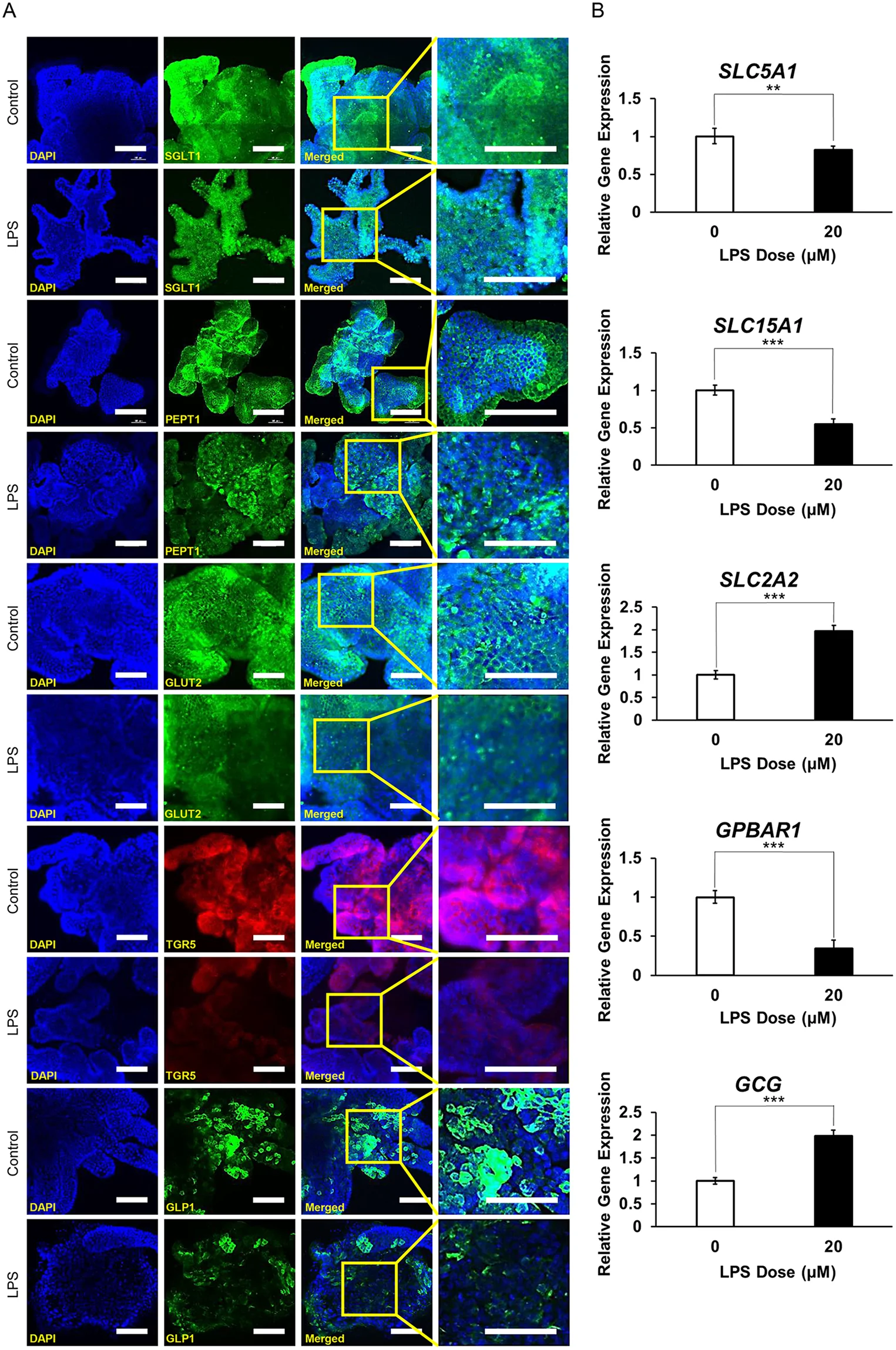
LPS-induced alterations in intestinal transport and enteroendocrine marker expression in pSIOs. (A) Immunofluorescence of pSIOs stained with DAPI (blue) to visualize nuclei and immunolabeled for SGLT1 (green), PEPT1 (green), GLUT2 (green), TGR5 (red), and GLP-1 (green). Representative untreated (upper rows) and LPS-treated (lower rows) organoids are shown. Untreated organoids exhibited distinct localization of SGLT1, PEPT1, GLUT2, and TGR5 along the epithelial membrane with strong expression and cytoplasmic GLP-1 expression in scattered enteroendocrine-like cells within the epithelial layer. LPS-treated organoids exhibited reduced expression intensity, irregular protein distribution, and partial loss of membrane localization. Enlarged views of the yellow boxed regions further highlight differences in expression patterns and subcellular localization between untreated and LPS-treated organoids. Images are representative of three independent biological replicates (n = 3), with at least five organoids analyzed for each treatment condition in each biological replicate. Scale bars: 50 μm. (B) qRT-PCR analysis showing significant alterations in nutrient transporter and enteroendocrine-related gene expression following LPS treatment. Decreased *SLC5A1* (SGLT1) and *SLC15A1* (PEPT1) expression and increased *SLC2A2* expression were observed after LPS exposure. Among enteroendocrine-related genes, GPBAR1 expression was markedly decreased, whereas GCG (the precursor gene of GLP-1) expression was significantly increased after LPS exposure.Data are presented as the mean ± SD from three biological replicates (n = 3); each biological replicate was analyzed using five technical replicates. **p < 0.01, ***p < 0.001.

In the untreated control group, the glucose uptake transporter SGLT1, the peptide transporter PEPT1, and the glucose efflux transporter GLUT2 exhibited continuous and uniform immunofluorescence signals primarily along the epithelial cell membrane. In contrast, in the LPS-treated group, these transporters exhibited an overall reduction in immunofluorescence signal intensity and uneven and discontinuous distribution. Consistent with the immunofluorescence findings, qPCR analysis revealed that the mRNA expression levels of *SLC5A1* (SGLT1) and *SLC15A1* (PEPT1) decreased 0.826-fold and 0.527-fold, respectively, following 20 μM LPS treatment. In contrast, the mRNA expression of *SLC2A2* (GLUT2) increased 1.299-fold compared with the control group.

The bile acid receptor, TGR5, exhibited normal expression patterns, with relatively uniform distribution throughout the cell membrane and cytoplasm in the untreated control group. However, after LPS treatment, its immunofluorescence signal intensity decreased, and an irregular distribution pattern was observed with weakened localization at the cell membrane. Consistently, qPCR analysis demonstrated that the mRNA expression level of *GPBAR1* (TGR5) decreased 0.345-fold in the 20 μM LPS-treated group compared with the control group.

Finally, *GLP-1* was expressed locally in the cytoplasm of a subset of cells within the epithelial layer in the untreated control group. In the LPS-treated group, a reduction in fluorescence intensity was observed, accompanied by structural disruption and fragmentation of GLP-1-positive regions. qRT-PCR analysis revealed that the mRNA expression level of *GCG* increased 1.978-fold in the 20 μM LPS-treated group compared with the control group.

### Transcriptome analysis and DEG identification

To identify transcriptional changes induced by LPS in pSIOs, differential expression analysis was performed using a cut-off criterion of *p* ≤ 0.05 and |log2 fold-change| ≥ 1. Significant upregulated and downregulated DEGs were identified in the LPS-treated group compared with the control group (Fig 6A). Analysis of the top 10 DEGs based on fold change identified the gene group with the most pronounced transcriptional responses to LPS (Fig 6B). The statistical significance and expression differences of DEGs were comprehensively analyzed using a volcano plot, and the top five genes showing the most pronounced expression differences were identified as *DUOX1*, *AQN-1*, *CCL26*, *BST2*, and *SFTPA1* (Fig 6C). Heatmap analysis and hierarchical clustering of the top 100 DEGs showed that the two groups are separated by treatment condition and that LPS treatment induces a distinct expression pattern compared to the control group (Fig 6D).

**Fig 6:**
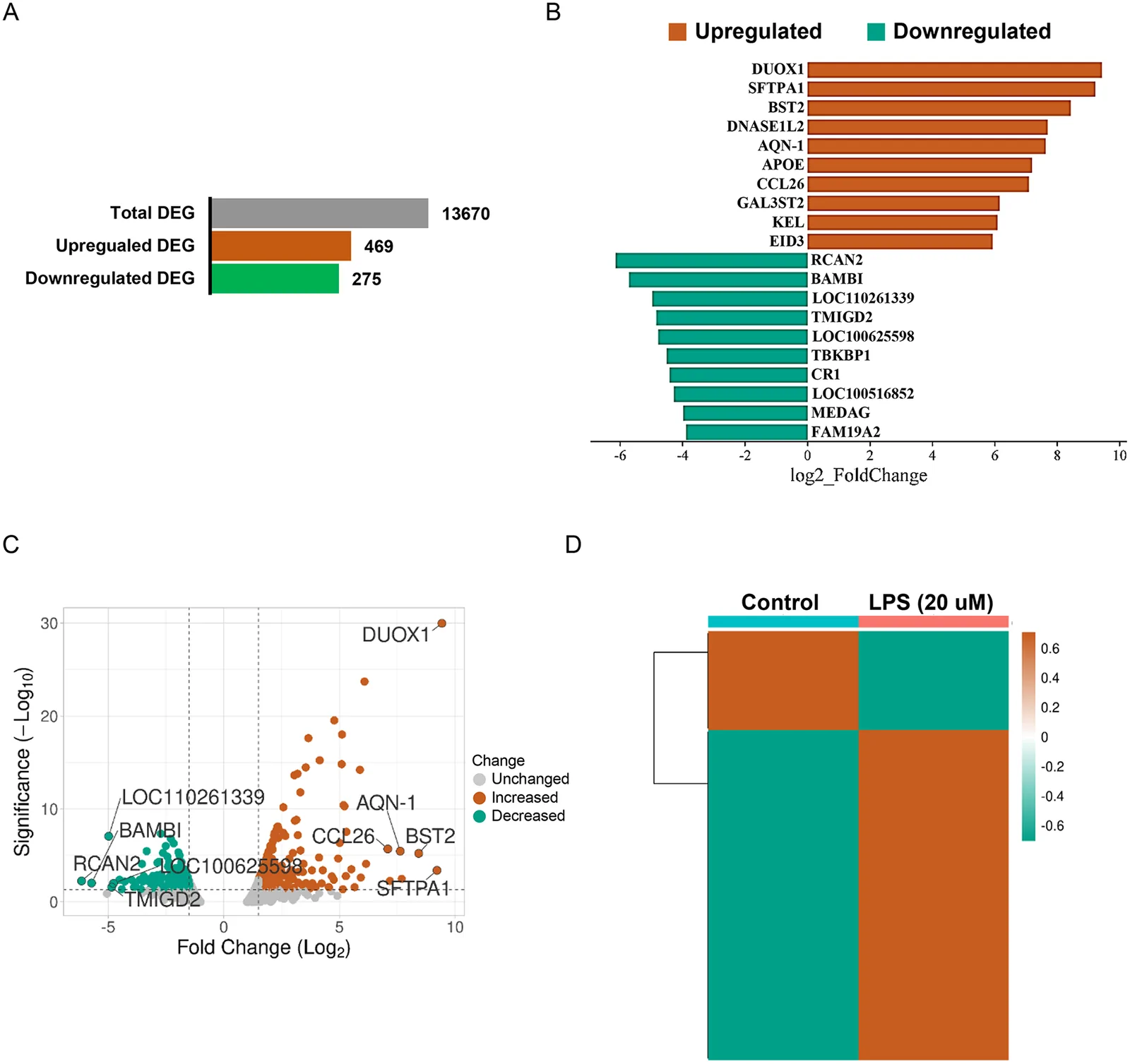
Identification of DEGs in pSIOs after LPS treatment. (A) Total number of upregulated and downregulated DEGs (*p* ≤ 0.05 and |log2 fold-change| ≥ 1). (B) Top 10 upregulated and downregulated DEGs ranked by fold change. (C) Volcano plot of DEGs with top five genes highlighted. (D) Heatmap and hierarchical clustering of the top 100 DEGs. RNA sequencing was performed using one pooled RNA sample generated from three organoid wells for each experimental group, resulting in one biological replicate (n = 1) per group. DEG, differentially expressed gene.

### GO and KEGG pathway enrichment in LPS-treated pSIOs

To elucidate the regulatory mechanisms of transcriptome changes induced by LPS stimulation in pSIO, we performed GO function enrichment analysis (BP, MF, and CC) and analysis of the top 10 KEGG pathways. In the BP category, the immune and host defense programs were the most prominent, including defensive responses to external stimuli, responses to other organisms and external biological stimuli, and immune activation regulatory processes (Fig 7A). In the MF category, the top 10 significantly enriched terms (*p* < 0.05) included chemokine activity, receptor-ligand activity, cytokine activity, and chemokine receptor binding (Fig 7B). In the CC category, the top 10 enriched terms (*p* < 0.05) included membrane and epithelial interface structures, including the apical plasma membrane, the plasma membrane region, and the apical part of the cell (Fig 7C). This suggests that LPS-responsive genes are preferentially localized to pathways operating at the cell surface and the apical epithelial compartment.

**Fig 7:**
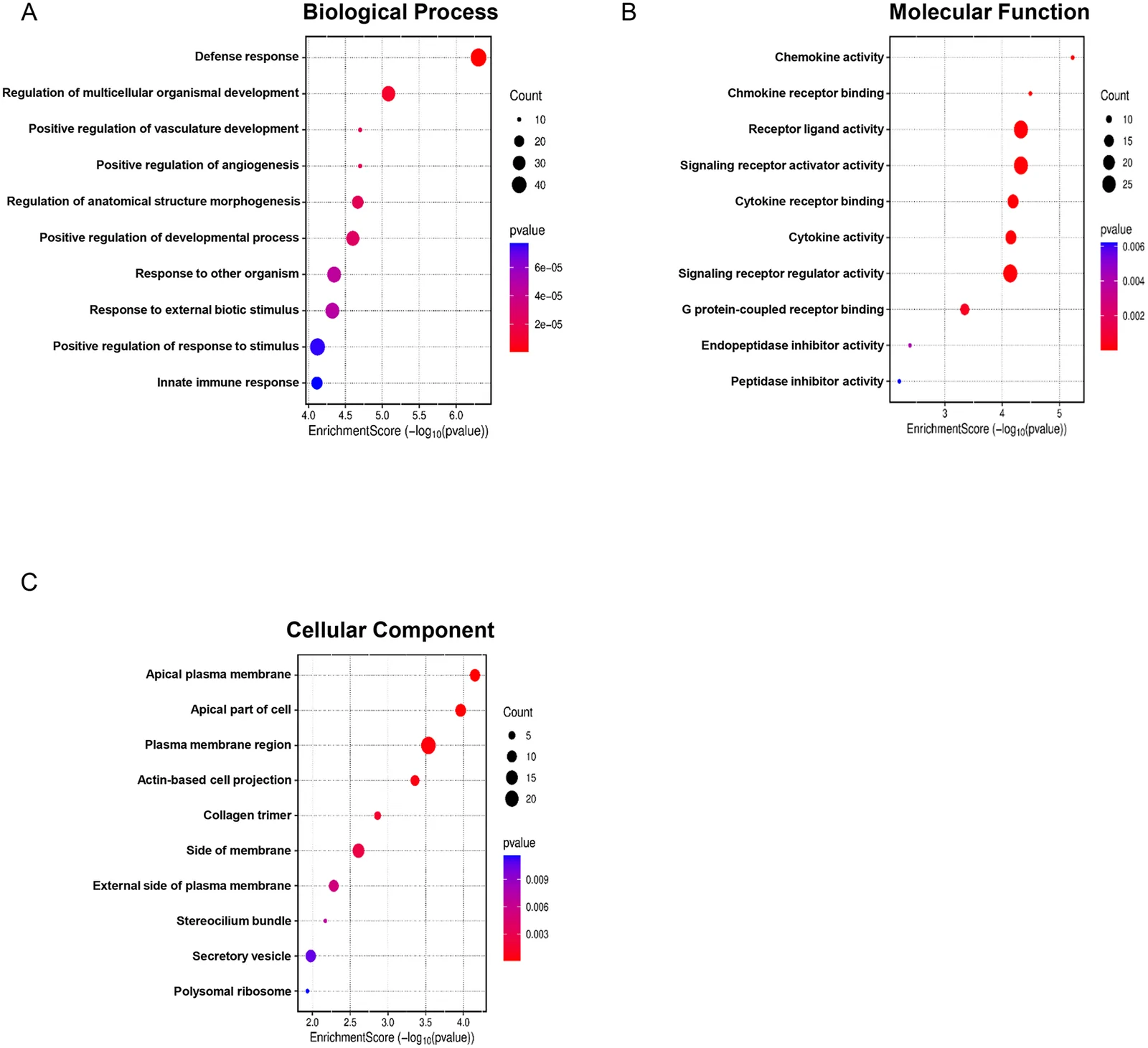
Gene Ontology (GO) enrichment analysis of DEGs in LPS-treated pSIOs. Top 10 significantly enriched (*p* < 0.05) GO terms in (A) biological process (BP), (B) molecular function (MF), and (C) cellular component (CC).

### KEGG pathway enrichment analysis in LPS-treated pSIOs

KEGG pathway enrichment analysis of DEGs in LPS-treated pSIOs identified immune and inflammatory signaling pathways as the dominant transcriptional responses (p < 0.05). Gene-pathway network analysis showed that a significant number of DEGs were involved in multiple pathways, forming close interactions between inflammatory signaling networks (Fig 8A). Analysis of the top 10 enriched pathways (Fig 8B) identified cytokine–cytokine receptor communications as the most prominent, followed by complement and coagulation cascades, viral protein interaction with cytokines and cytokine receptors, and the IL-17 signaling pathway. Other significantly enriched pathways were NF-κB signaling, TNF signaling, and other infection-related pathways.

**Fig 8:**
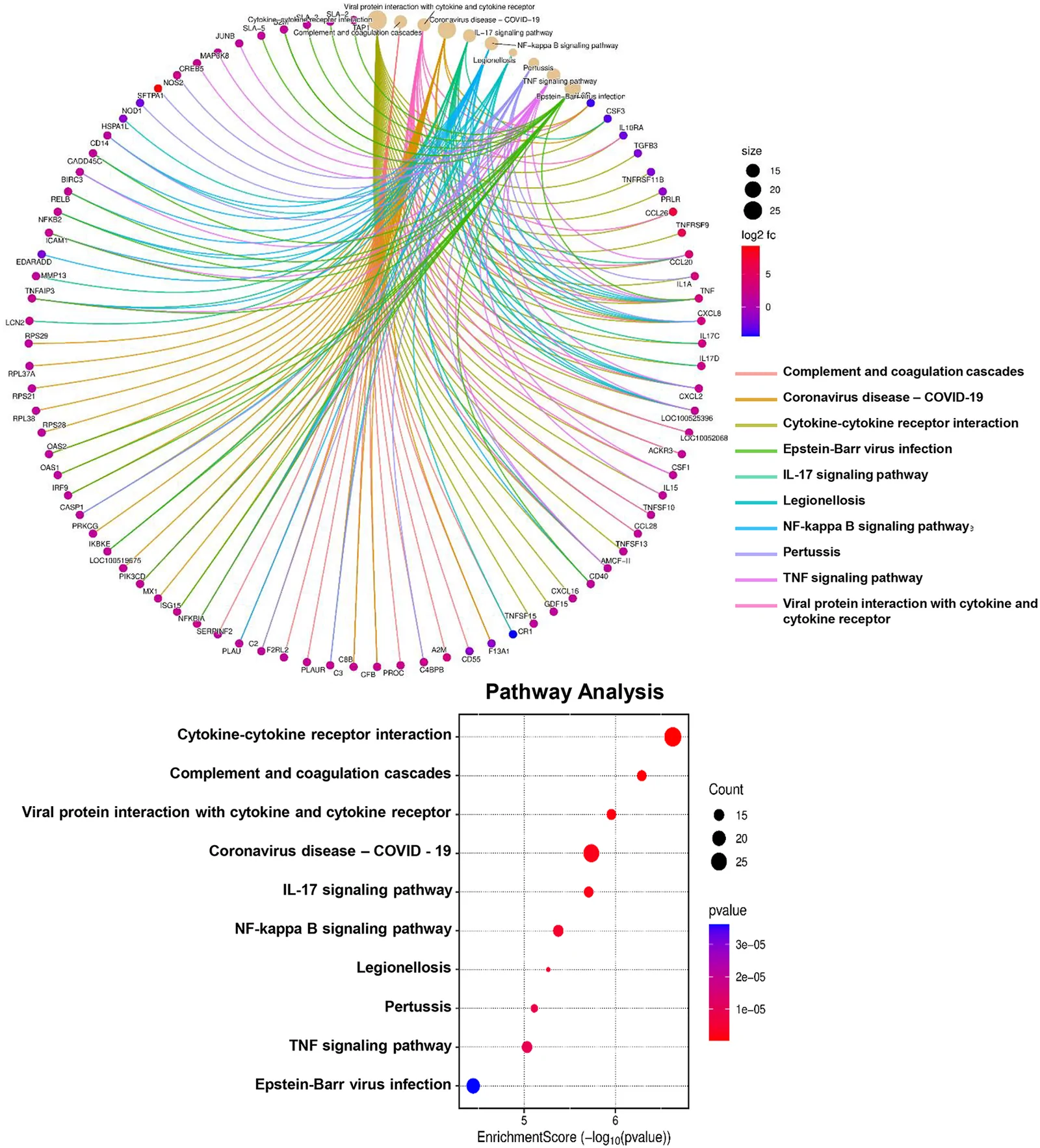
KEGG pathway enrichment analysis of DEGs in LPS-treated pSIOs. Top 10 significantly enriched pathways (*p* < 0.05) are shown. (A) Cnetplot showing specific genes in multiple enriched pathways. (B) Dot plot visualization of the top 10 enriched pathways.

## Discussion

Organoids are next-generation *in vitro* models that replicate the 3D architecture and cellular diversity of an organ. pSIOs are a robust model for studying intestinal inflammation because of their physiological similarity to the human intestine and experimental reproducibility. In this study, basal-out pSIOs derived from adult jejunum were used to evaluate epithelial inflammatory responses to LPS. LPS treatment induced organoid structural disruption and increased pro-inflammatory cytokine expression, accompanied by significant alterations in nutrient transport, receptor signaling, and endocrine function markers. These findings support pSIOs as a physiologically relevant *in vitro* model for investigating innate immune responses and inflammation-associated epithelial alterations.

In the present study, immunofluorescence analyses were performed using three-dimensional (3D) organoids to preserve the native epithelial architecture, cell–cell interactions, and spatial organization that closely resemble those of the in vivo intestine. Although organoid-derived two-dimensional (2D) monolayer systems provide improved antibody accessibility and facilitate more precise visualization of protein localization, they do not fully retain the three-dimensional structural complexity of the intestinal epithelium [30,35,43]. Therefore, we selected the 3D organoid model to evaluate protein expression within an intact epithelial context. Nevertheless, we acknowledge that the 3D configuration may limit antibody penetration and make precise subcellular localization more challenging than in 2D monolayer systems. This should be considered when interpreting the immunofluorescence findings.

Compared with the previous applications of porcine intestinal organoids, which have mainly focused on enteric infections, host–pathogen interactions, and intestinal epithelial biology, the present study expands their application by providing an integrated characterization of LPS-induced inflammatory responses. While earlier studies have often relied on evaluating a limited set of inflammatory markers or specific barrier proteins, we simultaneously evaluated epithelial barrier integrity, inflammatory cytokine expression, nutrient transporter regulation, enteroendocrine responses, and global transcriptomic alterations within a single experimental platform. This integrated approach provides a more comprehensive understanding of LPS-induced intestinal inflammation and supports the use of porcine small intestinal organoids as a physiologically relevant in vitro model for mechanistic studies and the evaluation of potential therapeutic or nutritional interventions. The physiological relevance of this model was further supported by the observation that pSIOs exhibited characteristics representative of diverse intestinal epithelial cell types. Elevated epithelial marker (*VIL1*, *LYZ*, and *CHGA*) expression suggests the presence of absorptive enterocytes, Paneth cells, and enterochromaffin cells, consistent with previous studies reporting that small intestinal organoids recapitulate multiple epithelial lineages observed *in vivo*, highlighting species-specific characteristics between human and porcine organoids [8,20,23,43,45]. Expression of stem cell markers (*LGR5, OLFM4,* and *SOX9*) confirms the maintenance of a stem cell population within pSIOs [46], supporting intrinsic self-renewal capacity [47–49]. Increased *MKI67* expression further indicates active proliferation, reflecting the rapid turnover typical of the intestinal epithelium [50]. Collectively, these results demonstrate that the pSIOs in this study possess both epithelial cell characteristics and stem cell-driven regenerative capacity.

Microscopic observation revealed that LPS treatment induced concentration-dependent morphological changes in pSIOs. LPS-untreated organoids maintained a typical budding structure and stable morphology, whereas structural stability gradually decreased with increasing LPS concentration. The findings indicate that increasing concentrations progressively compromise organoid structural integrity. Furthermore, PI staining revealed a concentration-dependent increase in cell death, with localized cell death at lower doses and widespread PI-positive signals at higher concentrations. These findings are consistent with those of previous reports that LPS simultaneously impairs morphological stability and cell survival of intestinal organoids. Similar fragmentation and increased apoptosis have been observed in a porcine small intestinal epithelial cell line (IPI-2I) [51] and human intestinal organoids following LPS treatment [52]. *In vivo* animal models further show that LPS induces mucosal barrier disruption, epithelial shedding, and cell-cell junction damage in porcine and rodent models [53]. Moreover, LPS downregulate E-cadherin, weakens cell–cell adhesion and compromises barrier integrity [54–56]. Immunofluorescence analysis showed continuous and uniform E-cadherin localization along lateral membranes in control organoids, whereas LPS-treated organoids exhibited markedly reduced E-cadherin intensity and discontinuous localization along cell-cell borders. Therefore, LPS stimulation disrupts the structural integrity of adherens junctions. qRT-PCR further revealed significant downregulation of *CDH1* (E-cadherin) and *TJP1* (ZO-1), the latter indicating disruption of tight junctions, leading to increased epithelial permeability [57–59]. E-cadherin mediates cell–cell adhesion, whereas ZO-1 stabilizes tight junction complexes [60–62]. Their concurrent downregulation indicates that epithelial barrier disruption involves coordinated impairment of multiple intercellular junctional complexes. This observed disruption further supports the applicability of pSIOs as an *in vitro* model that recapitulates intestinal barrier dysfunction. These findings are consistent with those of previous studies demonstrating that LPS-induced barrier dysfunction involves TLR4/NF-κB signaling, leading to decreased tight junction protein expression and increased permeability [55,63]. Collectively, these findings demonstrate the utility of the pSIO model as a reliable screening platform for identifying pathological mechanisms of LPS-induced barrier damage and for screening novel barrier-protecting compounds.

In this study, TLR4 expression increased in LPS-treated pSIOs compared with the control group. TLR4 recognizes LPS from gram-negative bacteria and initiates downstream inflammatory signaling [64–66]. The increased TLR4 fluorescence intensity following LPS stimulation shows that pSIOs partially possess functional innate immune sensing mechanisms that can recognize and respond to bacterial endotoxins. LPS also triggered an inflammatory response in pSIOs, as demonstrated by pro-inflammatory cytokine upregulation. Concurrent anti-inflammatory cytokine upregulation indicates activation of a compensatory anti-inflammatory response to maintain immune homeostasis [67]. In contrast, *TGF-β* expression exhibited a non-significant increase, reflecting that its expression and function can vary depending on the stimulation conditions, cell state, and tissue environment [68]. These results are consistent with those of previous studies demonstrating a dynamic balance between pro- and anti-inflammatory cytokines following LPS stimulation [52,69]. The multifaceted cytokine expression patterns identified in the pSIO model demonstrate its utility as a valuable *in vitro* platform for studying porcine intestinal inflammation and epithelial immune responses.

Transcriptome analysis further supported the LPS-induced inflammatory phenotype in pSIOs. Differential expression profiling identified a distinct set of LPS-responsive genes, with more upregulated than downregulated DEGs, indicating a predominantly activated transcriptional response. Notably, highly upregulated genes, including *DUOX1*, *DUOXA1*, and *BST2*, are associated with epithelial defense and inflammatory signaling, suggesting that LPS robustly activated a defense system for the outer layer of the organoids. Functional enrichment analysis indicated that LPS exposure in pSIOs triggers coordinated epithelial responses that extend beyond inflammatory signaling, including processes related to barrier maintenance, membrane-associated functions, and cellular adaptations to inflammatory stress [70]. Consistently, KEGG pathway analysis showed significant enrichment of immune- and inflammation-related pathways, including cytokine-cytokine receptor interaction, complement and coagulation cascades, viral protein interaction with cytokine and cytokine receptor, IL-17, NF-κB, and TNF signaling [71], and other infection-associated pathways, reflecting overlap between LPS-responsive genes and conserved host-defense pathways [72].

The effects of 20 μM of LPS on gene and protein expression and intracellular localization of key transporters, receptors, and hormones involved in nutrient absorption, metabolic regulation, and signaling were further analyzed. SGLT1 and PEPT1, responsible for nutrient absorption in the intestinal epithelium, are crucial in maintaining metabolic homeostasis [73,74]. The decreased signal intensity of these transporters and membrane fragmentation observed after LPS treatment in this study suggest that inflammatory stimuli inhibit nutrient absorption. This is consistent with the observed reduction in *SLC5A1* and *SLC15A1* gene expression and with previous studies showing that inflammation inhibits intracellular glucose and peptide uptake at the transcriptional level [75,76]. The decreased TGR5 signal intensity and loss of membrane localization observed after LPS treatment indicate disruption of bile acid receptor signaling within small intestinal organoids [77,78]. TGR5, a bile acid-sensing receptor, is involved in energy metabolism and immune regulation, and the transcriptional downregulation of *GPBAR1* (TGR5) identified in this study supports the disruption of this signaling axis under inflammatory conditions. Given the central role of bile acids in host-microbiota metabolic communication, TGR5 disruption can lead to dysregulation of the microbe-bile acid-host interaction network, ultimately leading to impaired metabolic homeostasis of the intestinal epithelium [79–83].

Notably, GLUT2, which is localized to the cell membrane and mediates glucose export, also exhibited downregulation and loss of membrane localization following LPS treatment, indicating that LPS-induced inflammation disrupts not only glucose uptake by the intestinal epithelium but also overall metabolic homeostasis through glucose transport systems and the epithelial barrier [84]. This result is consistent with those of previous studies reporting that LPS-induced inflammatory responses lead to metabolic imbalance in intestinal epithelial cells by disrupting glucose transport [85,86]. However, the mRNA expression of *SLC2A2*, which codes for GLUT2, was significantly upregulated after LPS treatment. This reflects a transcriptional compensatory response in which cells attempt to maintain metabolic function in response to LPS-induced physical barrier disruption and protein degradation, or metabolic reprogramming in response to inflammation [87]. The ‘asymmetric regulation’ pattern, characterized by the simultaneous upregulation of *SLC2A2* (GLUT2) and downregulation of *SLC5A1* (SGLT1), suggests that glucose influx and efflux pathways in the intestinal epithelium are disrupted by different mechanisms under inflammatory conditions.

The overall decrease in *GLP-1* expression in LPS-treated pSIOs suggests a decline in secretory capacity because of dysfunction of L-cells, which are a specialized intestinal epithelial cell subtype distributed within the epithelial layer of villus-like structures in organoids [88,89]. Considering that *GLP-1* is crucial in promoting insulin secretion and maintaining intestinal barrier integrity, this downregulation implies simultaneous impairment of metabolic regulation and intestinal defense mechanisms under inflammatory conditions [90–94]. The upregulation of *GCG*, the precursor gene encoding *GLP-1*, following LPS treatment may represent an adaptive or compensatory endocrine response to inflammatory stimuli, suggesting reprogramming of intestinal endocrine function under inflammatory conditions [95–98]. Collectively, these findings suggest that LPS-induced inflammatory conditions in intestinal epithelial organoids affect not only immune-related responses but also broader metabolic and functional networks, including nutrient transporters, bile acid receptor signaling, and intestinal endocrine regulation. Thus, inflammatory stimuli may lead to coordinated alterations in metabolic stability and signaling regulation within the intestinal epithelium.

Nevertheless, this study has some limitations. First, the analyses were performed at a single time point using only LPS as the inflammatory stimulus, which limits the generalizability of the findings to other inflammatory conditions. In addition, further functional validation, including time-course analyses and TEER measurements, will be necessary to strengthen the utility of this model.

## Conclusions

This study demonstrates that pSIOs derived from adult pigs effectively mimic LPS-induced epithelial structural damage and increased inflammatory cytokine expression. Disruption of nutrient transporters, bile acid receptors, and endocrine factors was observed at both transcriptional and protein levels, accompanied by structural disruption of intestinal barrier proteins. These results demonstrate the value of pSIOs as a species-specific *in vitro* platform that has the potential to reduce reliance on animal models while retaining key physiological characteristics. Although this study is limited to a single time point and a specific stimulus, future studies incorporating time-course analyses and additional functional assessments, such as TEER measurements, will further validate the utility of this model.

## Supporting information

S1 Table Primers used for gene expression analysis using quantitative RT-PCR. (DOCX)
