# Correction: Lipopolysaccharide-induced alterations in barrier integrity and inflammatory signaling in porcine small intestinal organoids

**DOI:** 10.1371/journal.pone.0362885

**Published:** 2026-11-12

**Authors:** Sumin Park, Boram Lee, Dahye Kim, Sheet Sunirmal, Min Gook Lee, Haesun Lee, Sun A. Ock, Jae Gyu Yoo

S1 Table was uploaded incorrectly. Please see the correct S1 Table here.

## Supporting information

S1 Table Primers used for gene expression analysis using quantitative RT-PCR. (DOCX)
