# Supplementary material for: Lipopolysaccharide-induced alterations in barrier integrity and inflammatory signaling in porcine small intestinal organoids
Source: PLoS One. 2026 Oct 1;21(10):e0359086. doi: 10.1371/journal.pone.0359086 (PMC13630212; doi:10.1371/journal.pone.0359086)
Supplement: Supplementary file 1 [file pone.0359086.s001.docx]

**Table 1.** Primers used for gene expression analysis using quantitative RT-PCR

| **No.** | **Gene name** | **Accession number** | **Forward (5ʹ–3ʹ)** | **Reverse (5ʹ–3ʹ)** | **Product size (bp)** |
| --- | --- | --- | --- | --- | --- |
| 1 | GAPDH | NM_001206359.1 | GTCGGTTGTGGATCTGACCT | AGCTTGACGAAGTGGTCGTT | 210 |
| 2 | VIL1 | XM_001925167.6 | CGACGGTGACTGCTACATAG | ATGCCCTGTTTGAAGTAGCC | 222 |
| 3 | LYZ | NM_214392.2 | CGGCTTCTCAGACAACATGA | AGTGCTTCCAACATTGTGGT | 220 |
| 4 | CHGA | NM_001164005.2 | ATGAATAAGGGGGACACCGA | TCAAAACACTCCTGGCTGAC | 101 |
| 5 | LGR5 | NM_001315762.1 | GAACAACATCAGTCAGCTGC | GACTGAAGGCTTCGCAAATT | 201 |
| 6 | OLFM4 | XM_003482903.4 | CGGTTTCCAATTTCACTGGC | TGAGCTGTGATTTCCAAGCG | 115 |
| 7 | SOX9 | NM_213843.2 | AAGACTCTGGGCAAACTCTG | TAATCCGGGTGGTCCTTCTT | 107 |
| 8 | KI-67 | XM_021073747.1 | GAAATCAGGGATCAGGAGGC | ATCACGTCTCCATGCTTCAG | 110 |
| 9 | TNF-α | NM_214022.1 | CCAATGGCAGAGTGGGTATG | AGGTTGACCTTGGTCTGGTA | 201 |
| 10 | IL-1β | NM_001302388.2 | CCGATGGTGTCTGTGATTGT | TGGGCTCTTCTTCAAAGACG | 112 |
| 11 | IL-8 | NM_213867.1 | CTGGCAAGAGTAAGTGCAGA | GGGTCCACTCTCAATCACTC | 105 |
| 12 | TGF- β | NM_214015.2 | TTAAAAGTGGAGCAGCACGT | TGACATCAAAGGACAGCCAC | 118 |
| 13 | IL-4 | NM_214123.1 | GGACTTGACAGGAACCTGAG | ATCGTCTTTAGCCTTTCCAAGA | 101 |
| 14 | IL-10 | NM_214041.1 | GGCCCAGTGAAGAGTTTCTT | GCTTGGCAACCCAGGTAAC | 101 |
