# Supplementary material for: Lipopolysaccharide-induced alterations in barrier integrity and inflammatory signaling in porcine small intestinal organoids
Source: PLoS One. 2026 Oct 1;21(10):e0359086. doi: 10.1371/journal.pone.0359086 (PMC13630212; doi:10.1371/journal.pone.0359086)
Supplement: Supplementary file 2 [file pone.0359086.s003.docx]

**Response to Reviewers: Manuscript ID: PONE-D-26-24948**

**Title: Lipopolysaccharide-induced alterations in barrier integrity and inflammatory signaling in porcine small intestinal organoids**

**General Response:** We sincerely thank the Editor and the Reviewers for their careful evaluation of our manuscript and for their valuable comments and suggestions. We have revised the manuscript accordingly. All changes have been highlighted using Track Changes.

**Journal Requirements:**

**1. Please ensure that your manuscript meets PLOS ONE's style requirements, including those for file naming.**

(Response) We have carefully reviewed the manuscript and all associated files to ensure full compliance with the PLOS ONE style requirements, including the formatting and file naming guidelines. The manuscript and all submission files have been revised accordingly.

Changes in manuscript: The title page and all submission files have been revised to comply with the PLOS ONE style and file naming requirements.

**2. To comply with PLOS One submissions requirements, in your Methods section, please provide additional information regarding the experiments involving animals and ensure you have included details on (1) methods of sacrifice, (2) methods of anesthesia and/or analgesia, and (3) efforts to alleviate suffering.**

(Response) We have revised the Materials and Methods section to include the requested information regarding the animal experiments. Specifically, we have added details on the method of euthanasia, including intravenous administration of T61 to minimize animal suffering, clarified that no anesthesia or analgesia was administered because tissue collection was performed only after euthanasia, and stated that small intestinal tissues were collected immediately after euthanasia. The requested information has been added to the Materials and Methods section (Page 5, Lines 174–179) as below.

“An LPS-induced pSIO model was established using 10-month-old adult pigs. All animal procedures were approved by the Institutional Animal Care and Use Committee of the National Institute of Animal Science (NIAS 2022-0569). Animals were euthanized by intravenous administration of T61 to minimize animal suffering. No anesthesia or analgesia was administered because tissue collection was performed only after euthanasia. Small intestinal tissues were collected immediately after euthanasia.”

**3. We note that the grant information you provided in the ‘Funding Information’ and ‘Financial Disclosure’ sections do not match. When you resubmit, please ensure that you provide the correct grant numbers for the awards you received for your study in the ‘Funding Information’ section.**

(Response) We have carefully reviewed the funding information and confirmed that the correct grant number for this study is PJ01671901. The Funding Statement has been revised accordingly to ensure consistency between the Funding Information and Financial Disclosure sections. Changes in manuscript: (Page 25, Lines 835-863).

“This work was supported by the Rural Development Administration (RDA), Republic of Korea, under the Cooperative Research Program for Agriculture Science and Technology Development (Project No. PJ01671901). The funders had no role in study design, data collection and analysis, decision to publish, or preparation of the manuscript.”

**4. Thank you for stating the following financial disclosure: [This work was supported by the Rural Development Administration (RDA), Republic of Korea, under the Cooperative Research Program for Agriculture Science and Technology Development (Project No. PJ01671901)]. Please state what role the funders took in the study. If the funders had no role, please state: ""The funders had no role in study design, data collection and analysis, decision to publish, or preparation of the manuscript."" If this statement is not correct you must amend it as needed. Please include this amended Role of Funder statement in your cover letter; we will change the online submission form on your behalf.**

(Response) We thank the editor for this helpful comment. In accordance with the journal's guidelines, **we have removed all funding-related text from the manuscript and included the amended Role of Funder statement in the cover letter**, as requested.

**5. Thank you for stating the following in the Acknowledgments Section of your manuscript: [This work was supported by a grant from the Rural Development Administration (PJ01671901). The authors gratefully acknowledge the financial support provided by the Korean government. We also extend our sincere gratitude to all individuals who contributed directly or indirectly to the completion of this research.] We note that you have provided funding information that is not currently declared in your Funding Statement. However, funding information should not appear in the Acknowledgments section or other areas of your manuscript. We will only publish funding information present in the Funding Statement section of the online submission form. Please remove any funding-related text from the manuscript and let us know how you would like to update your Funding Statement. Currently, your Funding Statement reads as follows: [This work was supported by the Rural Development Administration (RDA), Republic of Korea, under the Cooperative Research Program for Agriculture Science and Technology Development (Project No. PJ01671901).] Please include your amended statements within your cover letter; we will change the online submission form on your behalf.**

(Response) In accordance with the journal's guidelines, we have removed all funding-related statements from the Acknowledgments section. In addition, we have revised the Funding Statement to provide the complete funding information and the role of the funders. The Acknowledgments have been revised to include only a general expression of gratitude.

Changes in manuscript: (Page 25, Lines 826-828).

“The authors sincerely thank all individuals who contributed directly or indirectly to this study.”

**6. Please include captions for your Supporting Information files at the end of your manuscript, and update any in-text citations to match accordingly. Please see our Supporting Information guidelines for more information:** [**http://journals.plos.org/plosone/s/supporting-information**](http://journals.plos.org/plosone/s/supporting-information)**.**

(Response) We have added the captions for all Supporting Information files at the end of the manuscript and updated the corresponding in-text citations accordingly. In addition, the primer sequence table has been moved to the Supporting Information and is now presented as S1 Table.

Changes in manuscript: The in-text citation to S1 Table has been added to the Materials and Methods section (Page 8, Line 278), and the captions for all Supporting Information files have been added at the end of the manuscript (Page 46). “Primer sequences are listed in S1 Table.”

**7. If the reviewer comments include a recommendation to cite specific previously published works, please review and evaluate these publications to determine whether they are relevant and should be cited. There is no requirement to cite these works unless the editor has indicated otherwise.**

(Response) We carefully reviewed all reviewer comments and confirmed that no specific references were explicitly recommended for citation. Nevertheless, in response to Reviewer 1's suggestion to better position our study within the existing literature, we added several relevant references on porcine intestinal organoids where appropriate. Relevant references on porcine intestinal organoid research have been added to the Introduction and Discussion (Refs. 31, 36, and 40–44).

**Reviewer 1**

**(Comment 1) The authors have described known effects of LPS in connection with inflammatory processes and epithelial integrity in detail, however, they have not considered adequately to what extent such processes have already been described from other studies based on porcine organoids. This should be added either in the Introduction or in the Discussion of their results.**

**(Response 1)** We have revised both the Introduction and the Discussion to better discuss the current state of research on porcine intestinal organoids and to more clearly position our study within the existing literature.

In the Introduction, we have added a summary of previous applications of porcine intestinal organoids, including studies on enteric infections, host–pathogen interactions, intestinal epithelial biology, and organoid-derived two-dimensional monolayer systems. We also clarified that comprehensive characterization of LPS-induced inflammatory responses in porcine small intestinal organoids remains limited, thereby emphasizing the rationale and significance of the present study.

In the Discussion, we further compared our findings with previous reports and highlighted that, unlike previous studies focusing primarily on infectious disease models, our study provides an integrated characterization of LPS-induced inflammatory responses by simultaneously evaluating epithelial barrier integrity, inflammatory cytokine expression, nutrient transporter regulation, enteroendocrine responses, and global transcriptomic alterations. These revisions better clarify how our study differs from previous porcine intestinal organoid studies and highlight the novelty and contribution of the present work.

Changes in manuscript: The relevant text has been added to the Introduction (Page 4, Lines 131–169) and the Discussion (Page 19, Lines 672–685.

(Introduction)

“Recent studies have established porcine intestinal organoids as physiologically relevant models for investigating enteric viral infections, host–pathogen interactions, and intestinal epithelial biology (40-42). Furthermore, two-dimensional organoid-derived monolayer systems have been developed to facilitate barrier function assays and pathogen infection studies (43, 44). However, comprehensive characterization of LPS-induced inflammatory responses in porcine small intestinal organoids, including epithelial barrier disruption, nutrient transporter and receptor regulation, enteroendocrine responses, and global transcriptomic alterations, remains limited. To address this gap, the present study aimed to establish a porcine small intestinal organoid-based inflammatory model that integrates molecular, functional, and transcriptomic analyses to provide a comprehensive platform for investigating intestinal inflammation and evaluating potential therapeutic interventions.

In this study, pSIOs were treated with LPS to establish an intestinal inflammatory model. We analyzed transcriptomic and functional changes, including the expression of inflammation-related genes and the expression and localization of barrier-associated proteins, nutrient transporters, receptors, and enteroendocrine hormones. Collectively, these analyses enabled a comprehensive characterization of LPS-induced inflammatory responses in pSIOs and demonstrated the utility of this model as a physiologically relevant in vitro platform for investigating porcine intestinal inflammation and evaluating potential therapeutic interventions.”

(Discussion)

“Compared with the previous applications of porcine intestinal organoids, which have mainly focused on enteric infections, host–pathogen interactions, and intestinal epithelial biology, the present study expands their application by providing an integrated characterization of LPS-induced inflammatory responses. While earlier studies have often relied on evaluating a limited set of inflammatory markers or specific barrier proteins, we simultaneously evaluated epithelial barrier integrity, inflammatory cytokine expression, nutrient transporter regulation, enteroendocrine responses, and global transcriptomic alterations within a single experimental platform. This integrated approach provides a more comprehensive understanding of LPS-induced intestinal inflammation and supports the use of porcine small intestinal organoids as a physiologically relevant in vitro model for mechanistic studies and the evaluation of potential therapeutic or nutritional interventions.”

**(Comment 2) A further major comment deals with the question why the authors have used three-dimensional rather than two-dimensional tissues for their immunofluorescence approaches. From their pictures it is hardly possible to identify the localization of the different protein. The potential use of two-dimensional tissues obtained from the original organoids has been described in a number of studies.**

(Response 2) We appreciate the reviewer’s critical observation regarding the clarity of our immunofluorescence images. We agree that 2D monolayer systems offer distinct advantages for high-resolution imaging. We have revised the Discussion to clarify the rationale for using three-dimensional (3D) organoids for immunofluorescence analyses.

Specifically, we explained that 3D organoids were selected because they preserve the native epithelial architecture, cell–cell interactions, and spatial organization of the intestinal epithelium, thereby providing a more physiologically relevant context for evaluating protein expression. We also acknowledged that, although organoid-derived two-dimensional (2D) monolayer systems facilitate antibody accessibility and more precise visualization of protein localization, they do not fully retain the three-dimensional structural complexity of the intestinal epithelium.

Furthermore, we have discussed the limitation that the 3D configuration may restrict antibody penetration and make precise subcellular localization more challenging than in 2D monolayer systems. These revisions clarify the rationale for our methodological approach and acknowledge the limitations associated with 3D immunofluorescence analyses.

Changes in manuscript: The relevant text has been added to the Discussion (Page 19, Lines 662-671)

“In the present study, immunofluorescence analyses were performed using three-dimensional (3D) organoids to preserve the native epithelial architecture, cell–cell interactions, and spatial organization that closely resemble those of the in vivo intestine. Although organoid-derived two-dimensional (2D) monolayer systems provide improved antibody accessibility and facilitate more precise visualization of protein localization, they do not fully retain the three-dimensional structural complexity of the intestinal epithelium (31, 36, 44). Therefore, we selected the 3D organoid model to evaluate protein expression within an intact epithelial context. Nevertheless, we acknowledge that the 3D configuration may limit antibody penetration and make precise subcellular localization more challenging than in 2D monolayer systems. This should be considered when interpreting the immunofluorescence findings.”

**Reviewer 2**

**(Comment 1) The term 'lipopolysaccharide' is written in lowercase multiple times in the abstract. It is recommended that on first occurrence in abstract, it be uniformly standardized as the abbreviation LPS (with the full name preceding the abbreviation).**

(Response 1) The Abstract has been revised accordingly. The first occurrence has been standardized as “lipopolysaccharide (LPS)”, and the abbreviation “LPS” has been used consistently throughout the remainder of the Abstract.

Changes in manuscript: This has been revised in the Abstract (Page 2, Lines 28-38).

**(Comment 2) Lines 98–100 specify that “Organoids were cultured in IntestiCult™ medium supplemented with 20 μM LPS for 24 h at 37 °C in a 5% CO₂ atmosphere”. Please clarify the rationale for selecting 20 μM LPS as the working concentration. State whether this dosage was determined based on your preliminary experiments or cited from previous published studies.**

(Response 2) The rationale for selecting the 20 μM LPS concentration has now been clarified in the Materials and Methods section. The working concentration was determined based on our preliminary dose-response experiments using 5, 10, 20, and 30 μM LPS. Phase-contrast microscopy and DAPI/PI staining demonstrated a dose-dependent increase in structural damage and apoptosis following LPS treatment. While lower concentrations induced relatively mild changes, 20 μM consistently produced pronounced inflammatory and epithelial alterations while maintaining sufficient organoid integrity for subsequent molecular and functional analyses. In contrast, 30 μM caused extensive structural disruption and cell death, making it less suitable for downstream experiments. Therefore, 20 μM was selected as the optimal concentration for establishing the LPS-induced inflammatory model.

Changes in manuscript: The relevant explanation has been added to the Materials and Methods section (Page 6, Lines 197–203).

“Preliminary dose-response experiments were conducted using 5, 10, 20, and 30 μM LPS to identify the appropriate LPS concentration. The 5 and 10 μM concentrations induced minimal inflammatory responses with no apparent morphological changes, whereas 30 μM caused severe structural disruption accompanied by extensive cell death. In contrast, 20 μM reproducibly induced inflammatory responses with only partial morphological alterations and without extensive cell death. Therefore, 20 μM was selected for all subsequent experiments.”

**(Comment 3) All figures presented in the manuscript suffer from poor resolution and blurry display. Please replace all figures with high-definition, clear versions to meet the journal's image requirements.**

(Response 3) We thank the reviewer for this valuable comment. In response, we have replaced all figures in the revised manuscript with high-resolution versions to improve image clarity and meet the journal's image quality requirements. In addition, the font sizes of figure labels, axis titles, and annotations have been increased to improve readability and ensure that all text is clearly visible. All figures were regenerated from the original source files and exported at high resolution to provide clear visualization of images, graphs, and text.

Changes in manuscript:

All figures have been replaced with high-resolution versions, and the font sizes of figure labels and annotations have been increased to improve readability throughout the manuscript.

**(Comment 4) The manuscript only states that experiments were performed with at least three independent experiments in the statistical section, but the exact number of biological replicates is not clearly specified for each individual experiment, including qRT-PCR, DAPI/PI staining, immunofluorescence and RNA sequencing. Please supplement the number of biological replicates for all in vitro assays in the Materials and Methods section as well as all figure legends. Meanwhile, clearly distinguish biological replicates from technical replicates.**

(Response 4) We have revised the Materials and Methods section to clearly specify the number of biological and technical replicates for each in vitro assay. Specifically, for qRT-PCR, each independently processed organoid culture was considered one biological replicate (n = 3), and each biological replicate was analyzed using five technical replicates. For DAPI/PI staining and immunofluorescence analyses, experiments were performed using three independent biological replicates (n = 3), and at least five organoids were analyzed for each treatment condition within each biological replicate.

For RNA sequencing, RNA extracted from three organoid wells was pooled to generate a single RNA sample for each experimental group, resulting in one biological replicate (n = 1) per experimental group. While we acknowledge the limitation of lacking biological replicates for the RNA-seq analysis, this pooling strategy was carefully implemented to minimize intra-well variability and establish a stable, representative global transcriptomic profile. Importantly, the RNA-seq data was utilized primarily as an exploratory screening tool to map broad pathway alterations. To ensure the reliability of our results, all key transcriptomic findings—including the significant shifts in inflammatory cytokines, tight junction proteins, nutrient transporters, and enteroendocrine markers—were successfully cross-validated and confirmed using statistically powered, independent biological replicates (n = 3) via qRT-PCR and immunofluorescence assays. We believe this comprehensive validation robustly supports the biological reliability of our transcriptomic data despite the n = 1 design.

In addition, the corresponding figure legends have been revised to clearly indicate the number of biological replicates for each experiment and to distinguish biological replicates from technical replicates where applicable.

Changes in manuscript: The relevant explanations have been added to the Materials and Methods section (Pages 8–17, Lines 273–611), including the qRT-PCR, DAPI/PI staining, immunofluorescence staining, and RNA sequencing subsections. The corresponding figure legends (Figures 2, 3, 4, 5, and 6) have also been revised accordingly.

(qRT-PCR)

“For each experiment, organoids were independently cultured, treated, and subjected to RNA extraction, with each independently processed culture considered one biological replicate (n = 3). For each biological replicate, qRT-PCR was performed using five technical replicates. Relative expression levels were calculated using the 2^-ΔΔCt^ method with GAPDH as the endogenous control based on the mean Ct values of the technical replicates.”

(Apoptosis analysis (DAPI/PI staining)

“DAPI/PI staining was performed using three independent biological replicates (n = 3), and at least five organoids were analyzed for each LPS concentration in each biological replicate.”

(Immunofluorescence staining)

“Immunofluorescence staining was performed using three independent biological replicates (n = 3), and at least five organoids were analyzed for each treatment condition in each biological replicate.”

(RNA sequencing data processing and quantification)

“For RNA sequencing, total RNA extracted from three organoid wells derived from the same organoid preparation was pooled to generate a single RNA sample for each experimental group (n = 1 per group). Therefore, RNA sequencing was performed without independent biological replicates.”

(Differential gene expression analysis)

“Because the experiment included only one biological sample per condition (n = 1), variance estimation from biological replicates was not possible”

(Statistical analysis)

“Statistical analyses were performed using GraphPad Prism (version 5.0; GraphPad Software, San Diego, CA, USA). Data are presented as the mean ± standard deviation (SD) from at least three independent biological replicates, unless otherwise stated. Statistical significance between two groups was determined using an unpaired Student's t-test.”

(Figure 2.)

“Data are presented as the mean ± SD from three biological replicates (n = 3); each biological replicate was analyzed using five technical replicates.”

(Figure 3B.)

“Images are representative of three independent biological replicates (n = 3), with at least five organoids analyzed for each LPS concentration in each biological replicate.”

(Figure 3C.)

“Images are representative of three independent biological replicates (n = 3), with at least five organoids analyzed for each treatment condition in each biological replicate.”

(Figure 3D.)

“Data are presented as the mean ± SD from three biological replicates (n = 3); each biological replicate was analyzed using five technical replicates.”

(Figure 4A.)

“Images are representative of three independent biological replicates (n = 3), with at least five organoids analyzed for each treatment condition in each biological replicate.”

(Figure 4B.)

“Data are presented as the mean ± SD from three biological replicates (n = 3); each biological replicate was analyzed using five technical replicates.”

(Figure 5A.)

“Images are representative of three independent biological replicates (n = 3), with at least five organoids analyzed for each treatment condition in each biological replicate.”

(Figure 5B.)

“Data are presented as the mean ± SD from three biological replicates (n = 3); each biological replicate was analyzed using five technical replicates.”

(Figure 6.)

“RNA sequencing was performed using one pooled RNA sample generated from three organoid wells for each experimental group, resulting in one biological replicate (n = 1) per group.”

**(Comment 5) There are multiple errors and mismatches regarding Figure 4 and its legend. The main text clearly states that Figure 4 consists of two parts: Panel A shows TLR4 immunofluorescence results, and Panel B presents qRT-PCR data of pro- and anti-inflammatory cytokines. However, the current figure legend incorrectly describes non-existent subpanels: “Pro-inflammatory cytokines TNF-α, IL-1β, and IL-8 (A–C) and anti-inflammatory cytokines IL-4, IL-10, and TGF-β (D–F) (Lines 286-287), and completely omits the description of TLR4 immunofluorescence. Please revise the figure legend to accurately match the actual content of Figure 4.**

(Response 5) We thank the reviewer for this valuable comment. We have carefully revised Figure 4 to ensure complete consistency between the figure, its legend, and the corresponding description in the Results section. Specifically, the Figure 4 legend has been corrected to accurately describe Panel A as the TLR4 immunofluorescence analysis and Panel B as the qRT-PCR analysis of pro- and anti-inflammatory cytokines. The incorrect descriptions of the non-existent subpanels (A–F) have been removed, and a detailed description of the TLR4 immunofluorescence results has been added. In addition, the corresponding text in the Results section has been revised to maintain consistency with the updated figure and legend.

Changes in manuscript: The Figure 4 legend has been revised to accurately describe Panel A as the TLR4 immunofluorescence analysis and Panel B as the qRT-PCR analysis of pro- and anti-inflammatory cytokines. The corresponding description in the Results section (Page 13–14, Lines 468–493) has also been revised to ensure consistency between the figure, legend, and text.

“To evaluate the inflammatory responsiveness of pSIOs, organoids were treated with LPS (20 μM), and the TLR4 protein and cytokine gene expression were analyzed. Immunofluorescence staining showed TLR4 expression in both the control and LPS-treated groups, with significantly higher fluorescence intensity in the LPS-treated group (Figure 4A). To further characterize the inflammatory response, the mRNA expression levels of pro- and anti-inflammatory cytokines were analyzed by qRT-PCR (Figure 4B). The pro-inflammatory cytokines, tumor necrosis factor-alpha (TNF-α), interleukin (IL)-1β, and IL-8 (p < 0.01) and anti-inflammatory cytokines, IL-4 and IL-10 (p < 0.05) were significantly upregulated in the LPS-treated organoids, showing 2.61-fold, 3.61-fold, 3.65-fold, 12.15-fold, and 3.40-fold increases, respectively. In contrast, transforming growth factor beta (TGF-β) expression increased 1.41-fold but did not reach statistical significance.

“Figure 4. Cytokine gene expression changes in pSIOs following LPS treatment.

(A) Immunofluorescence staining of TLR4 in pSIOs after LPS (20 μM) treatment. Nuclei were stained with DAPI (blue), and TLR4 expression is shown in green. Representative images suggest higher TLR4 immunofluorescence in the LPS-treated group than in the control group. Images are representative of three independent biological replicates (n = 3), with at least five organoids analyzed for each treatment condition in each biological replicate. Scale bar = 100 μm. (B) qRT-PCR analysis of pro- and anti-inflammatory cytokines in pSIOs after LPS (20 μM) exposure. Pro-inflammatory cytokines TNF-α, IL-1β, and IL-8 were significantly upregulated in the LPS-treated group compared with the control group. Anti-inflammatory cytokines IL-4 and IL-10 also showed significantly increased expression, whereas TGF-β expression was increased but did not reach statistical significance following LPS stimulation. Data are presented as the mean ± SD from three biological replicates (n = 3); each biological replicate was analyzed using five technical replicates. *p < 0.05, **p < 0.01.”

**(Comment 6) Only representative immunofluorescence images are presented in Figure 3, Figure 4, and Figure 5. Please add semi-quantitative analysis of fluorescence intensity and corresponding statistical results to improve the objectivity and persuasiveness of the findings.**

(Response 6) We sincerely thank the reviewer for this valuable and constructive suggestion and fully agree that quantitative analysis of immunofluorescence images can provide additional information under appropriate experimental conditions.

However, in the present study, immunofluorescence was not intended to quantify protein abundance or fluorescence intensity. Instead, it was performed to visualize the spatial localization of proteins associated with epithelial barrier integrity (E-cadherin), inflammatory responses (TLR4), nutrient transport (SGLT1, PEPT1, and GLUT2), bile acid signaling (TGR5), and enteroendocrine function (GLP-1), as well as to evaluate LPS-induced structural alterations within intact three-dimensional organoids.

Because individual three-dimensional organoids inherently differ in size, morphology, orientation, and imaging plane, direct comparison of fluorescence intensity among organoids is technically challenging and may introduce substantial bias. Therefore, our conclusions were not based solely on immunofluorescence observations but were supported by complementary quantitative analyses, including qRT-PCR and transcriptomic analyses, which consistently demonstrated LPS-induced inflammatory responses and epithelial alterations.

Similar approaches have also been reported in previous organoid studies. For example, Sarvestani et al. (Nature Communications, 2021) used immunofluorescence to assess the subcellular localization and co-localization of E-cadherin and β-catenin in human ulcerative colitis-derived organoids, while transcriptomic analyses were performed as part of the comprehensive characterization of the organoid model. Likewise, Zietek et al. (Scientific Reports, 2015) used immunofluorescence to determine the expression and spatial localization of intestinal nutrient transporters (SGLT1, PEPT1, and GLUT2), the bile acid receptor TGR5, and the enteroendocrine marker GLP-1 in small intestinal organoids. Notably, the immunofluorescence findings for GLP-1 were complemented by Gcg (encoding GLP-1) mRNA expression analysis, which showed the same expression pattern and demonstrated that the physiological characteristics of the organoids were preserved in vitro.

Based on these considerations, we respectfully believe that the representative immunofluorescence images presented in this study provide appropriate support for demonstrating the spatial localization of biologically relevant proteins and LPS-induced structural alterations in intact three-dimensional organoids. Accordingly, we have clarified this rationale in the revised manuscript.

Sarvestani et al. (Nature Communications, 2021)

“To confirm the tight association of E-cadherin/β-catenin proteins, as well as significant differences in their sub-cellular co-localization, we performed dual IF staining in iHNOs and iHUCOs (Fig. 2g).”

Zietek, Tamara, et al.(Scientific reports, 2015)

“We first determined the expression and localization of some known solute carriers as well as of the bile acid receptor TGR5 in polarized SI organoids by immunofluorescent staining (Fig. 1a and Supplementary Fig. S1).”

“Immunofluorescent staining confirmed the presence of GLP-1- and GIP-containing EEC in SI organoids (Fig. 2a,e). It is established that the number of GLP-1-containing cells increases from proximal SI/duodenum to distal SI/ileum and vice versa for GIP-containing cells. When preparing organoids from different parts of the SI, we obtained the same expression pattern for the Gcg (encoding GLP-1) and Gip mRNA showing that physiology is preserved in vitro (Fig. 2b,f).”
